# The effect of water filter pitchers on the mineral concentration of tap water

**DOI:** 10.1111/jphd.12649

**Published:** 2024-11-06

**Authors:** Loai Wadea Hazzazi, Armando E. Soto‐Rojas, E. Angeles Martinez‐Mier, Hani M. Nassar, George J. Eckert, Frank Lippert

**Affiliations:** ^1^ Department of Oral Biology Faculty of Dentistry, King Abdulaziz University Jeddah Saudi Arabia; ^2^ Department of Biomedical and Applied Sciences Indiana University School of Dentistry Indianapolis Indiana USA; ^3^ Department of Dental Public Health and Dental Informatics Indiana University School of Dentistry Indianapolis Indiana USA; ^4^ Department of Restorative Dentistry, Faculty of Dentistry King Abdulaziz University Jeddah Saudi Arabia; ^5^ Department of Biostatistics and Health Data Science Indiana University School of Medicine Indianapolis Indiana USA

**Keywords:** dental caries, drinking water, fluoride, trace elements, water filter pitcher, water filtration, water purification

## Abstract

**Objectives:**

To investigate the effect of water filter pitchers on the concentration of different minerals in tap water.

**Methods:**

Nine water filter pitchers (A–I) were chosen based on consumer preferences and Amazon reviews. Each filter was tested for its ability to modify the concentrations of fluoride, calcium, magnesium, potassium, and sodium in tap water. Tap water samples were collected before and after filtration, at various intervals (1, 5, 10, 30, 50, 75, and 100 L) during filtration, and analyzed using an ion‐specific electrode (fluoride) and atomic absorption spectrometry (other minerals). Statistical analyses were conducted to compare filtered and unfiltered water mineral concentrations.

**Results:**

Water filter pitcher effect: Filters F (*p* < 0.001) and G (*p* = 0.030) decreased fluoride concentrations. All filters except I (*p* = 0.235) and H (*p* = 0.717) decreased calcium concentrations (*p* < 0.01). Filters E (*p* = 0.018), D (*p* = 0.014), and G (*p* = 0.010) decreased magnesium concentrations. Filters I (*p* = 0.028) and D (*p* = 0.009) increased potassium concentrations. Filter A (*p* = 0.002) increased sodium concentrations, while C (*p* = 0.034) decreased sodium concentrations. Effect of filter aging: All filters affected mineral concentrations over time but to varying extents. Filter G had the most pronounced effect on reducing mineral concentrations compared to all others. No filter was able to completely remove fluoride from tap water, contrary to the claims made by three manufacturers.

**Conclusions:**

The present study highlighted that water filter pitchers vary greatly in their ability to affect mineral concentrations in tap water during their use. Further research is needed to develop more effective water treatment solutions.

## INTRODUCTION

Community water fluoridation (CWF) involves the controlled addition of fluoride to public drinking water at concentrations ranging from 0.7 to 1.2 mg/L, a practice strongly endorsed by the Centers for Disease Control and Prevention (CDC) due to its proven effectiveness in preventing dental caries [1]. Since its implementation in the United States in 1945, numerous longitudinal studies have consistently demonstrated the significant role of CWF in reducing the prevalence of dental caries across populations [2]. Presently, approximately 75% of the U.S. population benefits from fluoridated public drinking water [3]. There is an extensive body of literature on longitudinal preventive effects of CWF on dental caries. A systematic review concluded that CWF is effective in preventing the prevalence of dental caries in children with a 35% reduction in decayed, missing, and filled deciduous teeth (dmf) and a 26% reduction in permanent teeth decayed, missing, filled and treated, compared to non‐fluoridated areas. Additionally, the review identified a 15% increase in caries‐free children in their deciduous dentition and a 14% increase in their permanent dentition due to CWF [4]. Moreover, CWF is cost‐effective, providing substantial economic benefits through reduced dental treatment costs and improved oral health outcomes.

However, public confidence in tap water safety has been shaken by incidents such as the lead poisoning crisis in Flint, Michigan [5, 6]. This has led to a notable shift towards bottled water consumption and the widespread adoption of water filtration and purification systems (WFPS) in households [7, 8]. Between 2013 and 2018, bottled water consumption surged, particularly among Black and Hispanic populations, reflecting growing concerns about the quality and safety of tap water [9]. This trend is further highlighted by a study showing that 15.1% of U.S. adults consider their tap water unsafe, 39.0% believe bottled water is safer, and 25.9% dislike the taste of tap water. These negative perceptions are most common among younger adults, Non‐Hispanic Black and Hispanic individuals, those with lower education and income, and renters. As a result, these groups are more likely to consume bottled water and sugar‐sweetened beverages (SSBs) instead of tap water [10].

While improving public perceptions of tap water safety and taste could help reverse this trend, the growing reliance on bottled water carries unintended consequences. One significant concern is the potential loss of the benefits provided by CWF, which is crucial for preventing dental caries. For instance, a study conducted in Indianapolis, Indiana, found that only two of 92 sampled bottled water brands had fluoride levels comparable to CWF, raising concerns about the dental health implications of widespread bottled water use [11]. The introduction of water filtration systems further complicates the landscape.

The WFPS market in the U.S. has nearly doubled over the past 5 years and is expected to continue growing, driven by consumer concerns about tap water safety and the desire for improved water quality [12]. The market offers a wide array of options, ranging from cost‐effective water pitchers and tap‐mount filters to advanced reverse osmosis and whole‐house systems.

Among consumers, water filter pitchers stand out as a popular choice due to their simplicity, affordability, and portability. The CDC defines water filter pitchers as “pitchers that are filled from the top and have built‐in filters that water must pass through before being poured out for drinking or other use.” These filters often feature replacement cartridges filled with activated carbon and ion‐exchange resin [13]. Some manufacturers describe a three‐step filtration process: an initial mesh barrier to capture debris, activated carbon granules to enhance taste by removing mercury and chlorine, and ion exchange resin to trap copper, zinc, and cadmium ions [14]. However, despite their widespread use, there is limited research on the impact of these filtration systems on fluoride concentrations in tap water.

At present, the American Dental Association (ADA) has granted its Seal of Acceptance to only two water filter pitchers and one tap‐mount filter, deeming them effective at filtration without fluoride removal [15]. Nonetheless, the absence of ADA endorsement does not preclude other WFPS from effectively maintaining fluoride levels, as the ADA's Seal of Acceptance program remains voluntary. Research on WFPS's impact on fluoride concentrations in tap water is scant, outdated, and yielded conflicting results, with no dedicated studies assessing the effects of water filter pitchers on fluoride concentrations in tap water [16, 17, 18, 19, 20].

Therefore, the focus of this study was to provide evidence on the effect of water filter pitchers commercially available in the USA on fluoride concentration in tap water. Beyond fluoride, other minerals in water, such as calcium and magnesium, play vital roles in oral health. Calcium and magnesium, for instance, can enhance tooth strength through remineralization [21, 22, 23, 24]. Additionally, research suggests that calcium may partially compensate for inadequate fluoride levels in tap water for caries prevention [25]. A study involving school children also found an inverse relationship between caries experience and salivary potassium, while salivary sodium showed a positive association with dental caries [26]. Therefore, understanding how these filters affect not only the retention of fluoride, but also other beneficial minerals is essential for overall oral health.

The study tested several hypotheses regarding the effectiveness of water filter pitchers in reducing fluoride, calcium, magnesium, sodium, and potassium concentrations in tap water. The null hypotheses (H_0_) were as follows: (a) there is no difference between water filter pitchers in their ability to reduce the fluoride concentration in tap water; (b) there is no difference between water filter pitchers in their ability to reduce the concentrations of calcium, magnesium, sodium, and potassium in tap water; (c) the ability of water filter pitchers to reduce the fluoride concentration in tap water does not change during their use; and (d) the ability of water filter pitchers to reduce the concentrations of calcium, magnesium, sodium, and potassium in tap water does not change during their use.

## MATERIALS AND METHODS

### Selection of water filter pitchers

Due to the extensive and competitive water filter market, which features numerous established and emerging companies, we focused on studying the most commonly used water filter pitchers among U.S. consumers for this study. We utilized online platforms like Amazon, a widely trusted source for consumer purchases, to determine which water filter pitchers are the most popular and highly reviewed. Additionally, we referred to consumer guides to ensure our study focused on filters predominantly used by the U.S. population. Local department stores were avoided due to their potential regional exclusivity, as Amazon provides broader accessibility across the country. After conducting our search, nine water filter pitcher models (*N* = 9) were selected for this study.

In our selection process, we explored different models offered by each brand and included these variations in our study. Details displayed on the outer packaging and accompanying information, as well as any claims regarding the filter's ability to remove fluoride, were recorded. Upon purchase, relevant information for each filter was documented, including the maintenance required (e.g., filter material/pad renewal) and the expected duration of optimal performance before deterioration (Table 1). Among the nine pitchers studied, one model (PUR) has received the ADA Seal of Acceptance, indicating their efficacy in filtering water without removing beneficial fluoride [15].

**TABLE 1:** Studied water filter pitchers and their characteristics.

Code	Brand	Product/model	Filtration process	Filter replacement	Pre‐use instruction	Effect on fluoride*	Effect on calcium, potassium, magnesium, and sodium*
A	Brita	Everyday pitcher, standard filter, (36050)	Activated carbon granules Ion exchange resin	Every 40 gallons/151 liters	Rinse 15 s with cold water Fill then empty the pitcher 3 times before use	Does not remove fluoride	“Preserves certain healthy minerals in water”
B	Pure	7 cup pitcher filtration system	Activated carbon granules Ion exchange resin	Every 40 gallons/151 L	Rinse 15 s with cold water Fill then empty the pitcher one time before use	Does not remove fluoride	“Preserves certain healthy minerals in water”
C	Epic water filter	Pure water filtration jug	Proprietary blend of water filtration media within three activated coconut carbon	Every 150 gallons/every 2–5 months	Fill then empty the pitcher twice before use	Removes more than 98.4% of fluoride	No statement about removal/retention
D	Drink Soma	Soma 10 cup pitcher	Activated coconut shell carbon Charcoal	Every 40 gallons/every 2 months	Soak the filter with cold water for 15 min then rinse for 10 s	No statement about fluoride removal/retention	No statement about removal/retention
E	Clear_2_O	Gravity water filtration pitcher (GRP200)	Nano alumina fibers onto microglass filaments creating a non‐woven filter media with a strong electropositive charge (+) that removes sub 1‐micron contaminants via electro‐adsorption, not just mechanical filtration	Every 60 gallons	Rinse few seconds with cold water Fill then empty the pitcher 3 times before use	No statement about fluoride removal/retention	“Preserves certain healthy minerals in water”
F	Clearly filtered	Gravity‐Fed Water Pitcher model (CF‐PRF)	3 stages of filtrations First stage: woven mesh screening layer Second stage: granulated coconut carbon layer Third stage: proprietary composite shell	Every 100 gallons	Priming the filter by attaching the filter to a priming bag and running the water till it fills the bag then forcing the water out of the filter (to be repeated three times)	Removes more than 99.54% of fluoride	(no information provided)
G	Zero water	10 cup ready‐pour pitcher	5 stages ion exchange	Every 25–30 gallons	Clean with warm water and soap then rinse	Removes more than 99% of fluoride	Removes calcium, potassium, magnesium, and sodium up to 100%
H	Brita	Stream model (36238)	Activated carbon Proprietary dual‐layer filtration technology	Every 40 gallons/151 L	Rinse 15 s with cold water Fill then empty the pitcher one time before use	Does not remove fluoride	“Preserves certain healthy minerals in water”
I	Brita	Long last ^+^ model (OB06)	Patented pleated filter and proprietary active filtering agents in a housing, made without BPA	Every 120 gallons/every 6 months	Rinse 15 s with cold water Fill then empty the pitcher one time before use	Does not remove fluoride	“Preserves certain healthy minerals in water”

* Information gleaned from product packaging or manufacturer's website.

The study was conducted from October 2023 to January 2024 and the filters were stored at ambient conditions at the Oral Health Research Institute (OHRI) of the Indiana University School of Dentistry.

### Water filtration and sample collection

Due to the potential for fluctuations in tap water fluoride concentrations, fluoride levels in the water were analyzed in a prior study, confirming that the fluoride levels are approximately 0.7 mg/L [27]. Subsequently, for each pitcher filter, the pitcher was removed from the package, and the filter was installed in the pitcher. Cleaning instructions for the filter and the procedure of running water for a specified duration before using the filter were followed in accordance with the manufacturers' instructions, as outlined in Table 1. Water sample collection commenced immediately after the recommended running time for each filter. Samples (20 mL) were collected after 1, 5, 10, 30, 50, 75, and 100 L of water had passed through the filter. Additionally, unfiltered tap water was collected immediately prior to and after each pitcher has been studied. Consequently, nine samples were collected for each filter test and stored under ambient conditions until analysis. This process was repeated for each filter.

### Fluoride analysis

The fluoride concentration of all samples was determined by using a fluoride ion‐specific electrode (Orion #96–909‐00) as described by Martínez‐Mier et al. [28]. For each water sample, 1 mL of total ionic strength adjustment buffer II (Fisher Scientific) was added to a 1 mL aliquot of the water sample in a fluoride‐free polyethylene vial (7‐mL vial; Fisher Scientific). After that, the solution was mixed using a vortex mixer and placed under the electrode. Finally, the millivolt reading of each sample was compared to a standard curve to obtain the fluoride content values.

### Calcium, magnesium, potassium, and sodium analyses

Mineral contents were determined using an atomic absorption spectrometer (ICE 3000 series‐Thermo, England) equipped with background correction (a deuterium lamp) as well as cathode lamps at a wavelength of 422.7, 285.2, 589.0, and 766.5 nm suitable for the analysis of calcium, magnesium, sodium, and potassium, respectively. The applied concentration of the standard solutions covered the measurement range of the analytical method, which was characterized by the linearity of the calibration curve [29, 30]. All samples were prepared for analysis using polyethylene vials (7‐mL vial; Fisher Scientific). For calcium analysis, 1 mL of lanthanum chloride was added to all samples. All water samples were tested at a volume to ensure that all measured concentrations fell in the measurement range that was determined previously (calcium—0.25; magnesium and potassium—0.05; sodium—0.20 mL).

### Statistical analysis

Mineral concentrations of the unfiltered water samples collected immediately prior to and after filtration were averaged. For the purpose of comparing filters to unfiltered samples, all filtered samples were averaged. Differences in mineral concentrations between filtered and unfiltered water were tested for each filter using one‐sample *t*‐tests. One‐way analysis of variance (ANOVA) was used to compare the tested filters for differences in percent changes in each mineral. A two‐sided 5% significance level was used for all tests.

## RESULTS

The average concentrations of each mineral in unfiltered tap water during the experimental phase were as follows: 1.1 fluoride, 62.2 calcium, 28.4 magnesium, 9.6 potassium, and 36.0 ppm sodium.

### Filter characteristics

The most common filtration mechanism was activated carbon (six filters), followed by ion exchange (three filters), used alone or in combination with activated carbon (Table 1). Filters used up to five stages of filtration. Filter replacement intervals varied between every 25–30 to every 150 gallons (95–114 to 568 L). Filters C, F, and G claimed to remove fluoride to at least 98.4%, whereas other filters either made no claim to that effect or stated that they did not affect water fluoride concentrations. Only filter G claimed to remove calcium, magnesium, potassium, and sodium.

**FIGURE 1:**
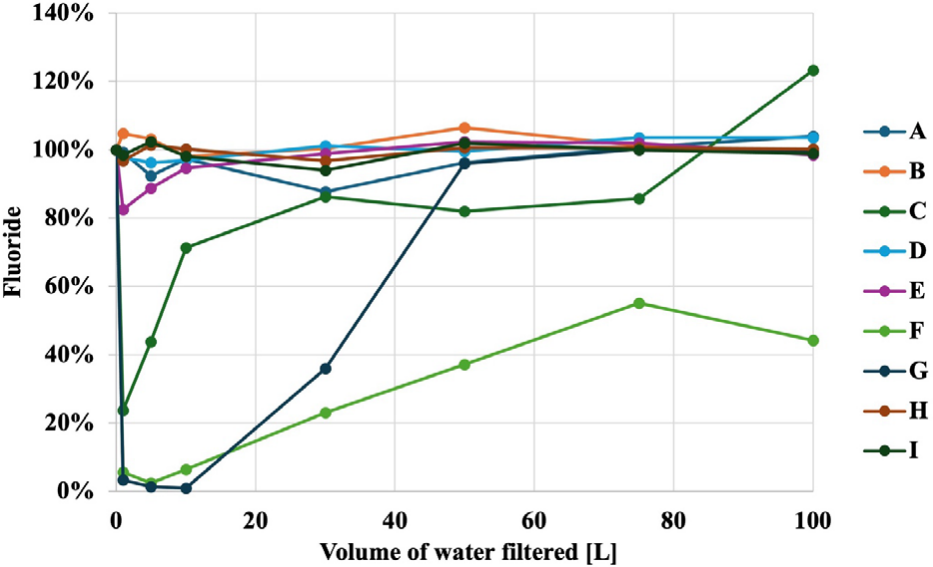
Fluoride concentration as a function of water filter pitcher and filtration volume. [Color figure can be viewed at wileyonlinelibrary.com]

**FIGURE 2:**
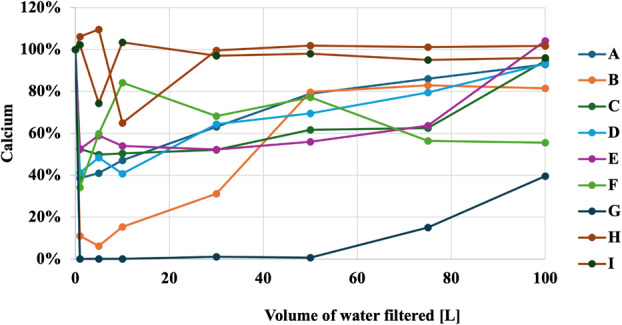
Calcium concentration as a function of water filter pitcher and filtration volume. [Color figure can be viewed at wileyonlinelibrary.com]

**FIGURE 3:**
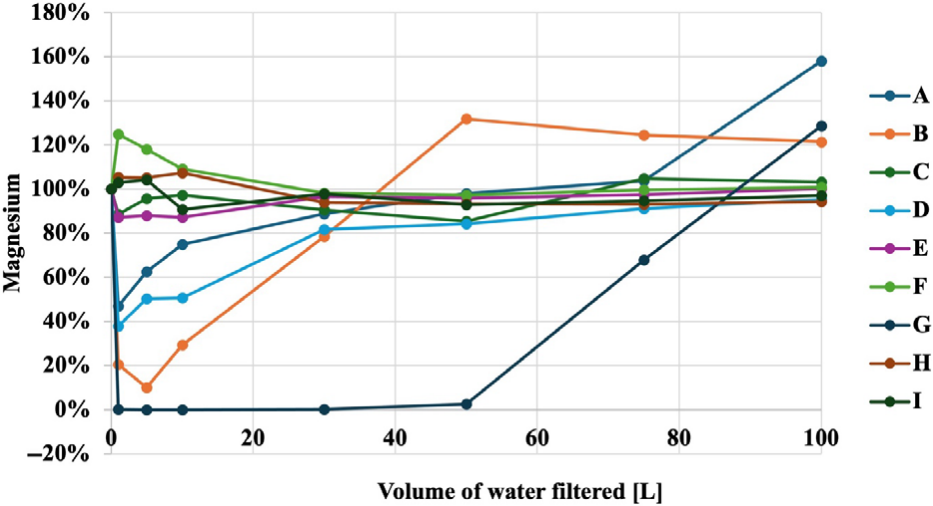
Magnesium concentration as a function of water filter pitcher and filtration volume. [Color figure can be viewed at wileyonlinelibrary.com]

**FIGURE 4:**
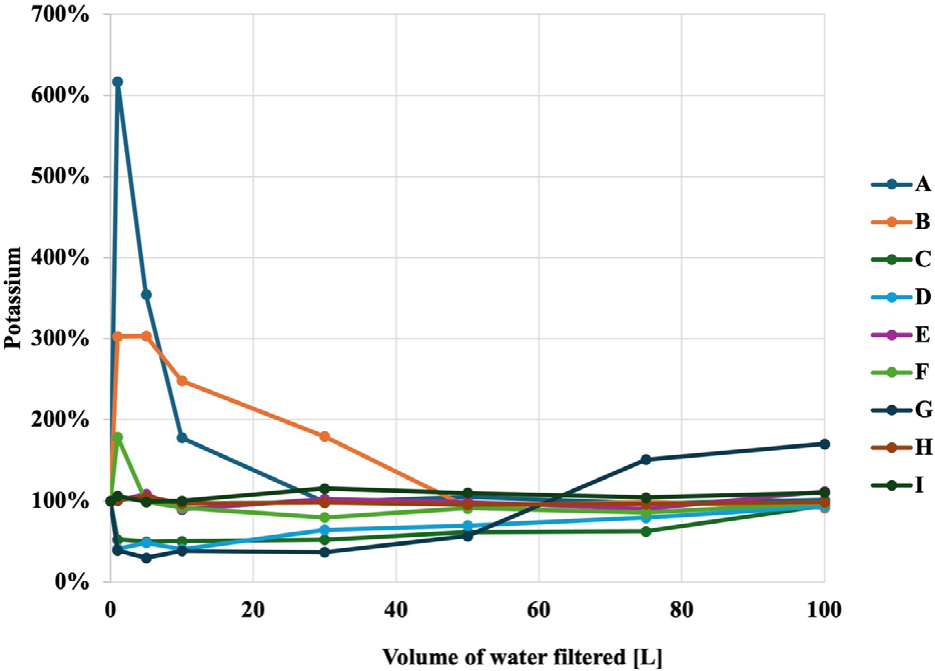
Potassium concentration as a function of water filter pitcher and filtration volume. [Color figure can be viewed at wileyonlinelibrary.com]

**FIGURE 5:**
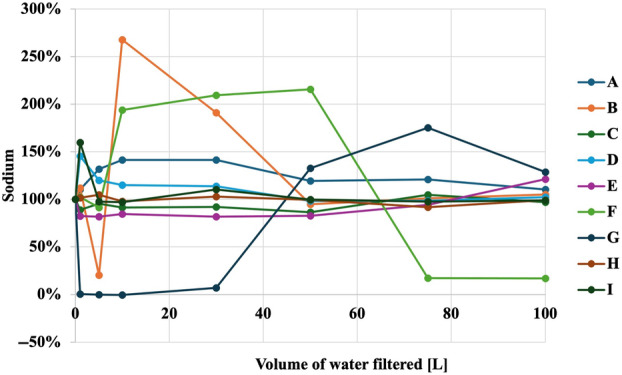
Sodium concentration as a function of water filter pitcher and filtration volume. [Color figure can be viewed at wileyonlinelibrary.com]

Figures 1, 2, 3, 4, 5 show the mineral concentrations by water filter pitchers and amount of water filtration volume. To allow for better comparisons of filter effects on mineral concentrations, unfiltered water samples were set at 100% and mineral concentrations of filtered samples were calculated in comparison to unfiltered samples.

Both filters F (*p* < 0.001) and G (*p* = 0.030), resulted in a significant decrease in fluoride concentrations. All filters, except I (*p* = 0.235) and H (*p* = 0.717), resulted in a significant decrease in calcium concentrations (*p* < 0.01). Filters E (*p* = 0.018), D (*p* = 0.014), and G (*p* = 0.010) demonstrated a significant decrease in magnesium concentrations. Filters I (*p* = 0.028) and D (*p* = 0.009) were notable for significantly increasing potassium concentrations. Moreover, filter A (*p* = 0.002) showed an increase in sodium concentrations, while C (*p* = 0.034) significantly decreased sodium concentrations.

### Effect of filter aging

#### Fluoride

Filter F had the biggest impact as fluoride concentrations did not return to baseline values during the experimental phase (Figure 1). Filters C and G exhibited significant reductions in fluoride concentrations initially, but both did not show an effect after 100 or 50 L, respectively.

#### Calcium

Filter G had the biggest impact as calcium concentration did not return to baseline values during the experimental phase (Figure 2). Filter F showed an initial reduction in calcium concentration followed by sudden increase but failed to return to baseline after 50 L of water filtration. All other filters resulted in an initial decrease in calcium concentration followed by a gradual increase, before reaching baseline values or values close to it.

#### Magnesium

Filter G had the biggest impact as magnesium concentrations did not return to baseline values during the experimental phase (Figure 3). Filters A, B, and D showed a significant reduction in magnesium concentration initially, but did not show an effect after 30, 50, and 30 L, respectively.

#### Potassium

Filter A initially increased potassium concentrations, but then displayed a gradual decrease over time (Figure 4). Similarly, D exhibited a significant fluctuation, with potassium levels rising sharply before declining rapidly. Filters C, D, and G showed similar trends, with potassium levels initially dropping before experiencing a sudden surge and subsequent stabilization.

#### Sodium

Filter G had the biggest impact on sodium concentration; however, concentrations returned to baseline values after 75 L (Figure 5). Filter B exhibited fluctuations in sodium levels, but these stabilized and returned to baseline values after 50 L. Filter F initially experienced a decrease in sodium levels, followed by a gradual increase, but subsequently dropped again, failing to return to baseline values after 50 L.

## DISCUSSION

According to the National Health and Nutrition Examination Survey (NHANES) 2007–2010 data [7], 33.7% of US adults use water treatment devices, including carbon filters, fiber filters, reverse osmosis units, neutralizers, chemical feed pumps, disinfection and softeners, and water filter pitchers. Although there are no specific data on water filter pitchers alone, they are likely the most used form of filtration along with faucet‐mount filters due to their comparatively low cost [31]. The change in mineral content after filtration has a major effect on drinking water. Therefore, the objective of the present study was to assess the impact of commercially available water filter pitchers on fluoride, calcium, potassium, magnesium, and sodium concentrations. To the authors' knowledge, this study is the first of its kind.

The results of the present study demonstrated that all filters affected the concentrations of at least some of the tested minerals in tap water to varying extents, thereby rejecting the null hypotheses a and b. Notably, Filter G emerged as the filter with the most pronounced impact (Figures 1, 2, 3, 4, 5). Filters F and C also displayed significant effects on fluoride, calcium, and sodium concentrations. In contrast, Filters A, B, and D exhibited diverse impacts on potassium and magnesium, with fluctuations observed throughout the filtration process, thus also rejecting the null hypotheses c and d. The interpretation of the present data is further complicated by the recommendations for filter replacement (Table 1). Users may also not be aware that filter replacement is warranted unless an indicator signals to the users that it is time to do so. For example, filter G affected fluoride concentrations for approximately half of its usage period (25–30 gallons), resulting in users being inconsistently exposed to fluoride. Filter C maintained its effectiveness in reducing fluoride concentrations longer than Filter G before eventually surpassing baseline levels. However, given that Filter C has a longer recommended replacement interval, its overall impact on fluoride exposure is likely to be less significant compared to Filter G.

The present results diverge from Jobson et al. [32] findings, where activated carbon filters significantly removed fluoride. The discrepancy may stem from differences in the number of water filter pitchers tested and the volume of water filtered. The present study investigated up to 100 L of water passing through a filter, whereas their study only tested up to 100 mL.

In contrast, Buzalaf et al. [33] findings were similar to the present ones. They found that most domestic activated carbon water filters do not remove fluoride. However, their study tested both new and old filters and collected only two samples of filtered and unfiltered water for analysis.

Konno [34] demonstrated that hollow‐fiber membrane filters or activated carbon filters used for domestic water filtration do not significantly remove fluoride from tap water. Although all filters in our study employed activated carbon, known to absorb chlorine, lead, and other contaminants, some brands incorporated ion exchange resins or other filtration media. Interestingly, we observed some filters exhibiting simultaneous increases in one element and decreases in another. For instance, Filter G showed a trend where calcium concentrations increased while sodium concentrations decreased over time. Similarly, Filter F displayed a gradual rise in sodium levels, accompanied by a notable reduction in potassium. We hypothesize that this phenomenon may be attributed to the proprietary materials utilized in these filters, acting as ion exchange materials.

The observed phenomena of filters exhibiting simultaneous changes in different elements, such as Filters G and F, present intriguing implications in the context of water filtration. Filter G displayed an increase in calcium concentrations over time, coinciding with a decrease in sodium concentrations, suggesting a complex interplay between the filtration mechanism and the chemical composition of the filtered water. Similarly, Filter F demonstrated a unique pattern, with sodium concentrations gradually increasing after the initial filtration, while potassium concentrations sharply dropped after filtering a certain volume of water. Lastly, several filters exhibited mineral concentrations that were initially lower but eventually higher than baseline values after 100 L of tap water passed through (e.g., Figure 1, C; Figure 4, G). There are multiple reasons that we believe are causing these effects on the minerals. One, the minerals were initially trapped inside the filter and then leached out again. Second, the filter became saturated. Third, it simply stopped working. These findings underscore the importance of understanding the dynamic interactions between filtration systems and the diverse array of minerals present in water. Further investigation into the underlying mechanisms driving these changes is warranted to optimize water filtration technologies and ensure the delivery of safe and nutritiously balanced drinking water.

In the present study, we focused on water filter pitchers, which are popular among consumers due to their portability and built‐in filtration systems. These filters are designed to remove undesirable contaminants such as heavy metals, lead, chlorine, and per‐ and polyfluoroalkyl substances (PFAS), aiming to enhance water taste and odor. While most of the brands we examined claimed to preserve fluoride along with other healthy minerals in the water, the present data on three filters (C, F, and G) is in contrast to these claims. Additionally, the impact of these filters on other minerals essential for oral health, including calcium, magnesium, potassium, and sodium was investigated. However, obtaining detailed information from the brands' websites proved challenging, as they often grouped these minerals under a generic category of “healthy minerals” without providing specific details. According to the authors' knowledge, no prior study investigated the effect of the pitcher water filters on the presently studied minerals. Thus, a comparison to previous studies is not possible. Moreover, some filters employed additional filtration processes beyond activated carbon, such as filtration media or screening barriers. Due to proprietary reasons, these processes were not disclosed, posing a challenge in interpreting the fluctuations observed in our results. Furthermore, the water samples were collected from a previously tested water source in Indianapolis with a known fluoride level. This presents a limitation of the study, as water from other cities may have different fluoride levels.

The present study had several strengths. First, it focused solely on popular water filter pitchers and compared different commercial brands. Second, the filters' aging effects on fluoride removal were investigated by tracking changes over time. Lastly, besides fluoride, we assessed other beneficial minerals in water, which some brands do not provide information on filtering. However, we also acknowledge that the present study also had limitations, including a relatively small sample size of only nine filter brands, a single 100 L observation for each filter brand, and limited access to unique filtration media blends, making it challenging to explain discrepancies in results.

For future research, it is imperative to conduct larger‐scale studies involving a wider range of water filter brands to further validate our findings. Additionally, exploring the long‐term effects of water filtration systems on fluoride and mineral levels in tap water could provide valuable insights into their efficacy and durability over time. Moreover, investigating the specific mechanisms underlying the observed changes in mineral levels, particularly those related to ion exchange features and proprietary filtration media, would enhance our understanding of how different filters impact water composition. Furthermore, studies focusing on the potential health implications of altered mineral levels in filtered water, particularly in relation to dental and overall health outcomes, would be beneficial. Lastly, transparency from manufacturers regarding the composition and performance of water filtration systems is crucial for consumers to make informed decisions about their water treatment options.

The shift from fluoridated tap water to bottled water carries significant economic consequences, notably the potential wastage of resources. The present study advocates for scientifically supported, cost‐effective water filtration alternatives that enhance public trust in tap water safety while preserving important minerals.

Additionally, the present study serves as a valuable resource for dental care providers and policymakers. It provides guidance on advising patients about their water consumption patterns and the benefits of specific filters. For manufacturers and policymakers, the present study emphasizes the importance of continual innovation in water filtration technologies and maintaining transparency about product features. This approach ensures that consumers can make well‐informed decisions that support their health and environmental sustainability. These insights encourage a more informed public and drive industry advancements to better meet consumer needs.

In conclusion, our study provides current evidence on the varying effects of water filter pitchers on the concentrations of fluoride and other minerals in tap water. While some filters effectively removed fluoride as claimed, others did not, and certain filters exhibited unexpected changes in mineral concentrations during their usage. Consumers should carefully research the capabilities and limitations of water filter pitchers, particularly regarding their impact on fluoride and essential mineral concentrations. Regular filter replacement is crucial, and users should choose filters with clear replacement indicators to ensure consistent performance. It is important to balance the removal of contaminants with the retention of other beneficial minerals like calcium and magnesium. Consumers should seek detailed information from manufacturers about filter technologies and monitor their water quality regularly.

## CONFLICT OF INTEREST STATEMENT

The authors declare no conflicts of interest.
